# Separating tectonic and climate signals in Holocene sea-level records using marine terraces in central Chile

**DOI:** 10.1038/s41598-026-43249-6

**Published:** 2026-03-13

**Authors:** Daniel Melnick, Julius Jara-Muñoz, Ed Garrett, Gaëlle Plissart, Roland Freisleben, Manfred R. Strecker

**Affiliations:** 1 Instituto de Ciencias de La Tierra, Universidad Austral de Chile, Valdivia, Chile; 2 Millennium Institute of Oceanography, Concepción, Chile; 3 Faculty of Civil Engineering, Institute of Geo and Environmental Sciences, Hochschule Biberach, Biberach an der Riß, Germany; 4 Department of Environment and Geography, University of York, York, UK; 5 Institute of Geosciences, University of Potsdam, Potsdam, Germany

**Keywords:** Climate sciences, Ocean sciences, Solid Earth sciences

## Abstract

Field observations of past sea-level variations are needed to validate models predicting future sea-level rise. Along tectonically active coasts, separating tectonic and non-tectonic sea-level components is challenging as both have similar amplitudes but necessary to decipher sea-level histories driven by climate forcing. Here, we present a new framework to decipher Holocene sea-level changes using marine terraces–geomorphic features formed by wave erosion of bedrock–mapped with high-resolution LiDAR data and numerical modelling. Applied to 266 sites along 500 km of central Chilean coast, we found that Holocene terrace elevations linearly correlate with Late Pleistocene terrace elevations, evidencing steady-state tectonics over the past 125,000 years. This proof of steady-state uplift allows subtracting tectonic components from Holocene elevations using uplift rates from Pleistocene terraces. We find that during the mid-Holocene, sea level reached 3.18 ± 0.15 m above modern elevation, only 0.33 m below glacial isostatic model predictions with 2·10^20^ Pa·s mantle viscosity. We validated this relationship by reproducing Holocene terrace elevations using a landscape evolution model and glacial isostatic sea-level curves. Our results suggest that accounting for millennial-scale vertical land motion rates that average many seismic cycles may improve future relative sea-level change projections, highlighting the potential of rocky-shore geomorphology for sea-level research along tectonically active coastlines.

**Supplementary Information:**

The online version contains supplementary material available at 10.1038/s41598-026-43249-6.

## Introduction

In the context of sea-level rise induced by modern climate change, assessing the hazards posed by coastal flooding requires validating existing models of potential sea-level variations with data from past sea-level positions. Along tectonically active coastlines, vertical land motion may either reduce or amplify relative sea level (RSL, sea level measured with respect to the adjacent coast) changes at decadal to millennial timescales, complicating the use of geologic and geomorphic markers of past sea-level positions to validate predictive models^1–3^. Tectonically active coastlines (coasts adjacent to tectonic plate boundaries or active geologic structures) represent 30% of the world’s coasts and commonly include shallow and deep deformation sources that will control the wavelength, rate, and polarity of vertical land motion. At millennial timescales, vertical land motion has been documented at a very wide range of rates exceeding 10 mm/yr of both uplift and subsidence^4^. Therefore, accurate sea-level histories along tectonically active coastlines require knowledge of vertical land-motion rates and evaluations of eventual changes over time.

A recent compilation of sea-level indicators including > 10,000 sites suggested that during the Holocene period (last 11.7 thousand years, ka), global mean sea level reached elevations higher than at present with a mean peak at 3.2 ka^5^. This peak has been referred to as the mid-Holocene highstand (HHS). Local sea-level reconstructions revealed that the HHS was associated with very different amplitudes reaching > 10 m and ages that peak between ~ 8 and 3 ka^5^. This large spatial and temporal variability has been attributed to equatorial ocean syphoning and continental levering^6^, which may explain the observed HHS at low-latitudes^7^. In addition to these oceanographic components, vertical land motion and glacial isostatic adjustments determine local sea-level histories and will thus influence their future evolution.

Understanding the role of local tectonic processes in driving RSL changes is instrumental for projecting future sea-level rise and its impacts on humankind, infrastructure, and ecosystems^8,9^. For example, the capacity of coastal wetlands (mangrove, tidal marsh and seagrass) to accumulate carbon is strongly controlled by RSL variations, which determine available accommodation space^10,11^. Therefore, an improved knowledge of the role of local tectonic processes on RSL changes is critical for projecting future coastal wetland carbon storage^12^. While vertical land motion may be quantified from coastal wetlands, this requires assessing various factors that may be difficult to quantify. This includes sediment input and compaction of detrital and biogenic sources as well as local and regional erosion processes^13,14^. In contrast, rocky coasts commonly border wetlands and sedimentary systems and offer alternative approaches to assess sea-level histories.

Rocky shores represent 69% of the world’s ice-free shorelines^15^, and of those 40% are associated with active tectonic structures^16^. Along rocky shores, sedimentary markers commonly used to derive sea-level index points^17^ are absent or limited to local embayments and pocket beaches, whereas marine terraces caused by wave abrasion may be continuously exposed along hundreds of kilometres^4^ and thus may serve as potential proxies to estimate variable sea levels of the past. The shoreline angle is a characteristic geomorphic feature of marine terraces that represents the position of mean sea level at the time of bedrock erosion^18^ and its elevation may be measured using topographic data and morphometric methods^19–21^.

Recent advances in the acquisition of high-resolution topography using Light Detection And Ranging (LiDAR) methods has allowed quantification of marine terrace elevations with high spatial resolution along continuous surveys spanning hundreds of kilometres^22–24^. Here, we present a novel method to separate the local tectonic contribution to RSL. First, we use LiDAR data to map Holocene marine terraces along 500 km of coast in central Chile and estimate shoreline angle elevations (Methods); In a second step, we subtract the effect of tectonic uplift using rates estimated over the same region from marine terraces of the last interglacial period^23^, allowing us to determine an average elevation of tectonically corrected RSL or mean sea level^25^ over the analysed stretch of coast. Finally, we compare these results with various sea-level curves predicted by Glacial Isostatic Adjustment (GIA) models to find the best-fitting model and reproduce shoreline elevations using a landscape evolution model of wave erosion and tectonic uplift.

### Tectonic and geomorphic setting

**Fig. 1:**
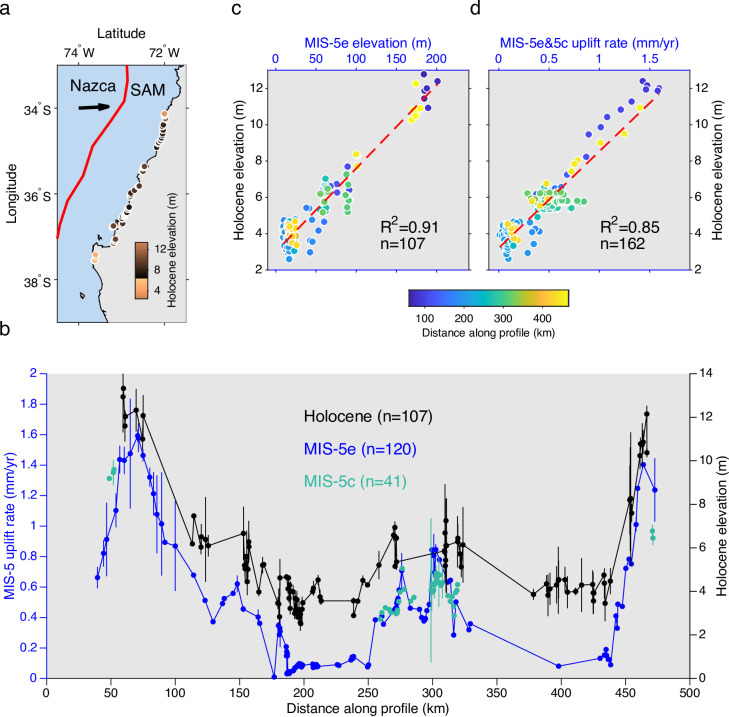
Empirical proof of steady-state coastal uplift in central Chile. (**a**) Tectonic setting of the Nazca-South America (SAM) plate boundary and location of Holocene terrace sites. Red line shows the Chile trench, black arrow plate convergence direction. (**b**) Coast-parallel profile showing locations of uplift rate determinations using MIS-5 terraces^23^ and elevations of Holocene terraces estimated in this study using a post-Maule earthquake datum. Dots show shoreline angle elevations, errorbars denote 2s uncertainty. (**c**) Relation between Holocene and MIS-5e terrace elevations from linear interpolation (see methods). Stippled red line shows linear regression. (**d**) Relation between Holocene terrace elevations and MIS-5 uplift rates from linear interpolation (see methods). R-Pearson correlation coefficient, n-number of points. Note the linear correlation suggesting steady-state tectonic uplift.

Subduction of the oceanic Nazca Plate beneath South America at 66 mm/yr controls most of the land-level changes along the Pacific coast of South America^26^. We focus on the Maule seismotectonic segment (34°S-38°S, Fig. 1a), which ruptured last in 2010 during the M8.8 Maule earthquake^27^. This megathrust earthquake was associated with a 500-km-long rupture zone and caused coastal land-level changes ranging between + 2.4 and −0.5 m^28^, and was preceded by a similar event in 1835 that also generated coastal uplift locally exceeding 2 m according to field measurements made by Darwin and FitzRoy^29^. Great megathrust earthquakes in the Maule segment are separated by centuries-long interseismic periods characterized by negative vertical motions (land subsidence) of up to ~ 10 mm/yr^29^, and decadal long postseismic periods associated with subsidence rates of up to ~ 20 mm/yr^30^.

The Maule segment is located at > 1000 km from the Patagonian icesheets and thus not significantly affected by contemporary post-glacial rebound^31^. Along the entire Chilean coast, geologic markers and GIA models predict the HHS reached up to 7 m (elevations reported above modern mean sea level) lasting between ~ 7.5 and 6 ka ago, based on a compilation of sea-level index points collected in sedimentary sequences^32^. The amplitude of RSL predicted by GIA models is a function of latitude, lithospheric thickness, continental mantle viscosity, and choice of ice model. Along the Maule coast (34°S-38°S, Fig. 1a), GIA models^31,33^ predict an HHS with peaks reaching 4.5 m between ~ 6.5 and 7.5 ka, depending on the choice of Earth rheology parameters. Higher peak elevations are predicted by models with a thicker lithosphere and lower mantle viscosity (Fig. S1).

**Fig. 2:**
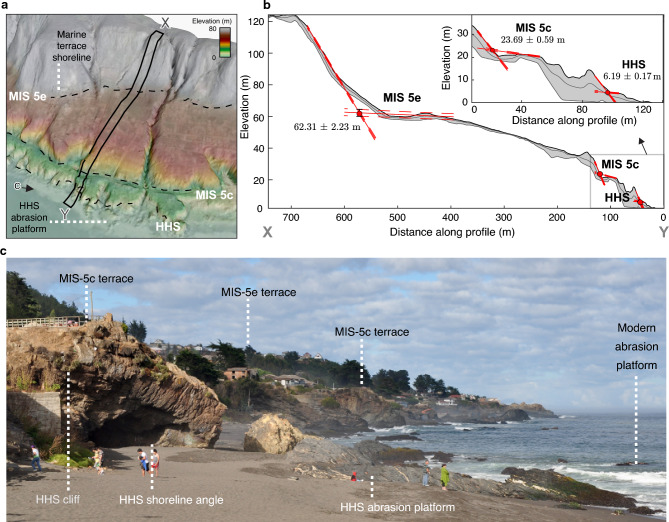
Bedrock marine terraces and geomorphic markers of past sea-level positions. (**a**) Oblique view of LiDAR terrain model showing marine terraces at Pelluhue (35.8°S). Stippled black lines represent inner edges of marine terrace associated with Marine Isotope Stages (MIS) 5e, 5c, and Holocene. HHS-Holocene Highstand. (**b**) Topographic swath profile (location in a). Red dots with error bars denote shoreline angle elevations estimated using TerraceM (see methods) by intersecting the paleo abrasion platform and paleo cliff (red lines). Inset shows zoom to lower levels. (**c**) Field view of marine terraces. Location and view of photograph in a.

Because sea levels were higher than at present during both the mid Holocene and last interglacial (also referred to as Marine Isotope Stage (MIS) 5e) periods, marine terraces of these highstands are ubiquitous along many coastlines, including central Chile (Fig. 2). Along 5000 km of the Pacific coast of South America, MIS-5e terrace elevations range from 5 to 210 m, with associated uplift rates of between 0.1 and 1.6 mm/yr, and a median of 0.22 mm/yr averaged over the past 125 ka^34^. These rates imply deformation wavelengths ranging from hundreds of kilometres and > 200 m amplitudes associated with deep-seated sources such as subduction of oceanic bathymetric anomalies, to short wavelength (< 10 km) linked with local crustal faults and folds^35^. Therefore, the marked gradients of vertical land motion along these coasts emphasize the need to collect densely spaced measurements and compare results at collocated sites to validate predictive models.

## Results and discussion

### Steady-state coastal uplift in central Chile at millennial-scale

Shoreline angle elevations in central Chile (Figs. 1 and 2) estimated using the TerraceM-2 software (a tool for quantitative assessment of marine terraces, see methods) and LiDAR data referred to a post-2010 earthquake vertical datum range between 6.2 and 204.1 m for MIS-5 terrace levels, and between 2.6 and 13.2 m and Holocene levels (Fig. 1b). We compared elevations of these two terrace levels at collocated positions by linear interpolation along a coast-parallel profile (Fig. 1b). We find significant linear correlations between Holocene and MIS-5e terrace elevations (Fig. 1c), and between Holocene terrace elevations and uplift rates derived from MIS-5e and MIS-5c terrace levels; these uplift rates were estimated using a sea-level curve for the southern hemisphere^36^ (Fig. 1d). These results suggest that analogous tectonic processes have been responsible for coastal emergence over the past 125 ka at constant uplift rates.

Tectonic uplift rates derived from MIS-5e terraces show mean and maximum along-coast gradients of 0.025 and 0.7 mm/yr per kilometre of coast, respectively (Fig. 1b). Therefore, comparisons of uplift rates derived from different terrace levels and dissimilar ages at non-collocated sites may suggest apparent changes in uplift rates over time. However, our dense database strongly indicates that such differences are better explained by spatial variations in uplift rates. Such bias is likely responsible for the ten-fold increase in uplift rates over 10^3^ to 10^5^-yr timescales inferred by Ref.^37^ in Central Chile and interpreted as a result of temporal earthquake clustering.

### Geomorphic estimate of the Holocene sea-level highstand peak elevation

**Fig. 3:**
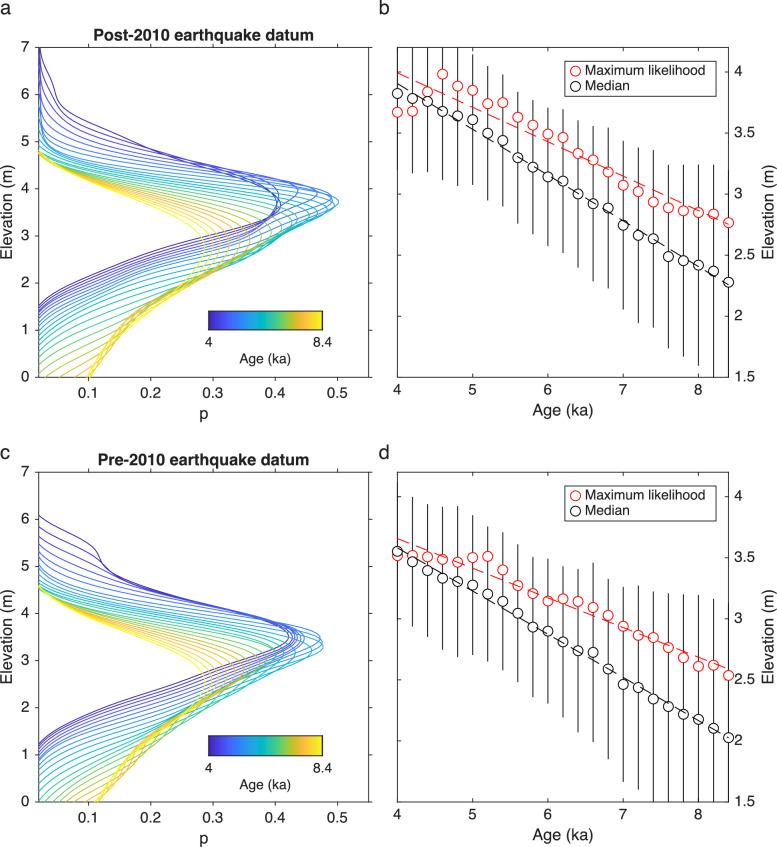
(**a**) Probability density functions of the Holocene highstand elevation estimated by subtracting the tectonic uplift component using MIS-5 uplift rates, for assumed highstand ages between 4 and 8.4 ka, using a pre-2010 earthquake vertical datum. (**b**) Maximum likelihood and median values of probability functions in a) with linear regression lines. Error bars represent interquartile range. Elevations above modern mean sea level. (**c**) Probability density functions as in a) but using a pre-2010 earthquake datum by accounting for coseismic uplift using the model of ref.^27^. (**d**) Maximum likelihood and median values of probability functions in b).

At each of our 106 Holocene terrace measurement sites, we estimated the elevation of the HHS by accounting for tectonic uplift using rates from adjacent MIS-5e terraces. First, we calculated the indicative meaning (see methods), which relates sea-level markers to mean sea level^38,39^ and ranges from -0.14 to 0.25 m at our sites (Fig. S2); secondly, we subtracted the corresponding values at each site. In a next step, we calculated the magnitude of tectonic uplift using MIS-5e uplift rates at each Holocene terrace site for an age range of 4 to 8.4 ka, based on absolute age determinations of Holocene sea-level markers along the Chile margin^32^, and subtracted these values from Holocene terrace elevations to obtain HHS peak elevations. We computed probability density functions of HHS elevations using all of the 106 sites (Fig. 3a), obtaining maximum likelihood elevations between 2.8 and 4.0 m, and median elevations between 2.3 and 3.8 m, with respect to a post-Maule earthquake vertical datum (Fig. 3b). The same analysis but referred to a pre-Maule earthquake datum (Fig. 3c) yielded maximum likelihood elevations between 2.5 and 3.5 m, and median elevations between 2.0 and 3.6 m (Fig. 3d).

**Fig. 4:**
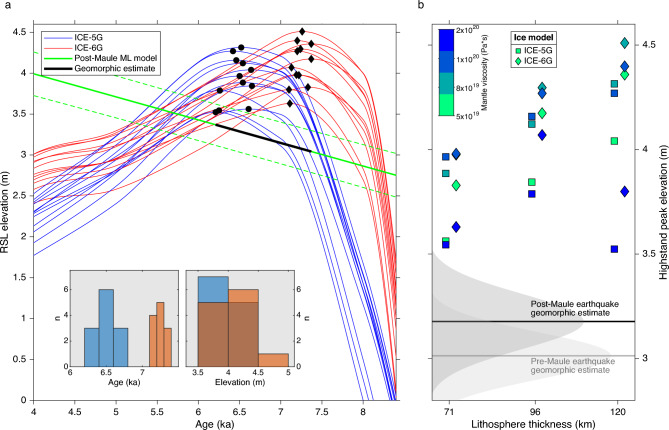
(**a**) Relative sea-level predicted by glacial isostatic adjustment (GIA) models. Dots and diamonds denote Holocene highstand peak elevations, with histograms in inset. Green line shows a maximum likelihood (ML) model based on estimates in Fig. 2b (referred to a post-2010 earthquake datum), stippled lines show 95% confidence bounds. Black line shows ML model in the age range predicted by GIA models, used to obtain the geomorphic estimate of the Holocene highstand. (**b**) Comparison of highstand elevations from our geomorphic estimate and GIA models with various Earth parameters. Note that all models slightly overpredict our geomorphic estimate.

To compare our geomorphic estimates with GIA models (see methods), we calculated the elevations and ages of HHS peaks predicted from a suite of models with different lithosphere thickness and mantle viscosity (Figs. 4a and S1). While ICE-5G models predict HHS elevations of 3.5 to 4.3 m between 6.2 and 6.7 ka, ICE-6G models suggest elevations from 3.6 to 4.5 m between 7.1 and 7.4 ka. Our geomorphic mean HHS elevation with respect to a post-Maule earthquake datum, and calculated with the maximum likelihood age model between 6.2 and 7.4 ka (range of HHS ages from both GIA models), is 3.17 ± 0.15 m, The geomorphic HHS elevation with respect to a pre-Maule earthquake datum is 3.01 ± 0.14 m. Probability density functions from HHS elevations estimated relative to pre- and post-Maule earthquake datums have 50% overlap (Fig. 4b) and mean values overlap within one standard deviation. The HHS estimate relative to a post-Maule earthquake datum is only 0.33 m below predictions from the ICE-5G model for a lithospheric thickness of 120 km and mantle viscosity of 2·10^20^ Pa·s (Fig. 4b). This viscosity falls within the lower bounds estimated from seismic-cycle models of the 2010 Maule earthquake^40,41^ and is slightly lower than global averages estimated from postglacial rebound models^42^.

**Fig. 5:**
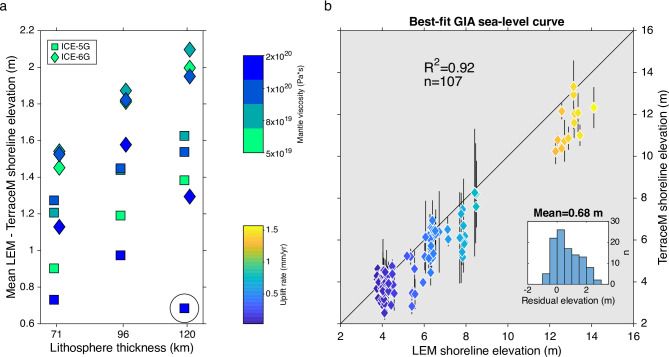
(**a**) Mean difference between shoreline angle elevations estimated from a landscape evolution model (LEM) using sea-level curves predicted by different GIA models and measured using the TerraceM method referred to a post-2010 earthquake vertical datum (see Methods). Black circle denotes best-fit model. (**b**) Comparison between TerraceM measured and LEM modelled Holocene shoreline elevations for the best-fit GIA sea-level curve and uplift rates estimated using MIS-5e terraces. Inset shows histogram of residual elevations. R-Pearson correlation coefficient, n-number of points. The black line denotes equal relation.

Using a Landscape Evolution Model (LEM) of marine terrace erosion under an oscillating sea level at a tectonically uplifting coast^19,43^ (see methods), we reproduced Holocene terrace elevations with sea-level curves predicted by GIA models and uplift rates from collocated MIS-5e terrace measurements. We searched for the sea-level curve from 24 different GIA models that minimized the mean difference between measured and modelled shoreline angle elevations (Fig. 5a), finding that the ICE-5G ice model with a lithospheric thickness of 120 km and mantle viscosity of 2·10^20^ Pa·s better fits our observations, with a mean of 0.68 m (using a post-Maule earthquake datum). The best-fitting modelled shoreline elevations are linearly correlated (r^2^ = 0.91) to measured shorelines (Fig. 5b); however, the asymmetric distribution of residuals (inset in Fig. 5b) shows that the LEM slightly overpredicts measured shoreline elevations. This probably results from the higher peak HHS elevation of the GIA model as well as with local processes not accounted for by the LEM such as local wave climate, bedrock erosion caused by sediment abrasion, and heterogeneous bedrock erodibility.

### Implications for projecting future sea-level rise along tectonically active coastlines

Our results suggest that rates of vertical land motion have been constant over the past 125 ka along the central Chilean coast and given the rather simple geodynamic boundary conditions of this subduction setting, these rates are not expected to change significantly over the next millennia. These millennial-scale rates represent an average of the coseismic, post-seismic and interseismic phases of many seismic cycles^8^, which along the studied seismotectonic segment of the margin (Maule segment) have a mean recurrence interval of 134 ± 87 years estimated over the past 4.5 ka^44^. Our HHS estimate with respect to a post- and pre-2010 earthquake datum is statistically undistinguishable, with a difference of 0.16 ± 0.21 m, although coseismic land-level changes during this earthquake ranged from + 2.4 and −0.5 m^28^. This HHS similarity is likely caused by the relatively large (n = 106) number of sites distributed over the entire 500-km rupture area that included the areas affected by coseismic coastal uplift and subsidence.

Therefore, using vertical land motion rates estimated over millennial timescales, which average out many seismic cycles, may help constraining the input parameters for GIA models at local scale. GIA models are crucial for projecting future RSL changes and obtaining accurate forecast scenarios for the safe operating space^45^ along the Earth’s coasts. Empirical data, such as the tectonically-corrected elevation of the HHS provided in this study, is needed to constrain the rheological input parameters of GIA models used in RSL forecast scenarios at local scale. Our results highlight the application of rocky-coast geomorphology for sea-level research along tectonically active coasts surveyed by high-resolution topography.

## Methods

### Analysis of wave-cut terraces

We mapped Holocene wave-cut terraces along the central Chilean coast using Digital Terrain Models (DTM) at 1-m resolution derived from airborne LiDAR data and the TerraceM-2 software^19^. The LiDAR data was collected between 2004 and 2007 at regional scale by the company Digimapas Chile and reprocessed using post-2010 earthquake GPS benchmarks in the EGM2008 datum. Using data from GPS buoys in the open ocean and collocated GPS sites and tide gauges, Ref.^46^ found that the difference between EGM2008 and mean sea level is 0.02 m at the coast facing the open ocean and between -0.1 and -0.18 in the Arauco Bay area (37°S) as a result of shallow bathymetry. Besides the Arauco Bay, the central Chilean coast is exposed to the open ocean and therefore the differences between EGM2008 and mean sea-level datums may be neglected. We adapted the indicative meaning algorithms of Ref.^38^ in MATLAB to estimate the elevation of modern analogues with respect to mean sea level along the study area following Ref.^39^.

The TerraceM-2 software (available at www.terracem.com) is a MATLAB Graphical User Interface designed to estimate the location and elevation of the shoreline angle using the surface morphology of marine terraces with DTMs and swath profiles. The shoreline angle is a geomorphic marker located at the intersection between the paleo-platform and paleo-cliff, and represents the maximum reach of the sea level during a highstand period that can be used to estimate vertical deformation and coastal uplift rates^18^. When the shoreline angle was found to be buried by colluvium deposits, TerraceM-2 allows estimating its elevation by extrapolating linear regressions of the exposed paleo-platform and paleo-cliff geomorphic features, accounting for error propagation in this intersection. In addition, TerraceM-2 includes a Landscape Evolution Modelling (LEM) toolbox, which is based on Ref.^43^, and simulates the dissipation of wave energy into rock erosion and cliff retreat under an oscillatory sea level and tectonic uplift. Model setups may be found in the codes provided in the online repository.

We used the coseismic deformation model of Ref.^27^ to refer the Holocene shoreline angle elevations to a pre-2010 earthquake datum (Fig. S4), in order to remove heterogenous deformation induced by this large earthquake and refer elevations to a late interseismic period datum.

We used the existing dataset of MIS-5e and 5c terraces^23^, which is based on a correlation of terrace levels and sea-level highstands using the absolute ages and a sea-level curve for the southern hemisphere^36^. Uplift rates (u) where estimated as:

$$\text{u }=\raisebox{1ex}{$\left(E-e\right)$}\!\left/ \!\raisebox{-1ex}{$T$}\right.$$

where *E* is the elevation of shoreline angles, *e* the position of the respective highstand and *T* the age of the terrace level. Uplift rate errors are estimated following^47^ as:

$$Se\left( u \right)^{2} = u^{2} \left( {\left( {{\raise0.7ex\hbox{${\sigma^{2} H/}$} \!\mathord{\left/ {\vphantom {{\sigma^{2} H/} {H^{2} }}}\right.\kern-0pt} \!\lower0.7ex\hbox{${H^{2} }$}}} \right) + \left( { {\raise0.7ex\hbox{${\sigma^{2} T/}$} \!\mathord{\left/ {\vphantom {{\sigma^{2} T/} {T^{2} }}}\right.\kern-0pt} \!\lower0.7ex\hbox{${T^{2} }$}}} \right)} \right)$$

where σH is the error in relative sea-level defined as:

$$\sigma H = \sqrt {\sigma E^{2} + \sigma e^{2} }$$

where *σE* is the age uncertainty in the sea-level curve, *σE* is the error of the shoreline angle measurement and *σe* is the 12 m uncertainty of the highstand elevation^36^. We used an arbitrary uncertainty of 7 ka for the duration of the MIS-5e highstands (*σE*) following Ref.^23^.

### Glacial isostatic adjustment models

We compared our estimate of the mid-Holocene sea-level highstand elevation with glacial isostatic adjustment (GIA) models of RSL. The inputs of GIA models are an ‘ice model’ providing global ice sheet changes and an ‘Earth model’ that accounts for the rheological properties of the solid Earth. We assume only radial variations of the Earth structure with different values of upper/lower mantle viscosity and lithospheric thickness. We calculated RSL at 1 ka intervals using the ICE-5G^31^ and ICE-6G^33^ ice models, which are the most commonly used in GIA models, and 12 Earth models that account for rheologies commonly inferred for central Chile based on seismic-cycle models^40,41^. We used upper mantle viscosities of 5*10^19^ Pa*s, 8*10^19^ Pa*s, 10^20^ Pa*s, and 2*10^20^ Pa*s, and a lower mantle viscosity of 10^22^ Pa*s, with lithospheric thicknesses of 71, 96, and 120 km. Upper mantle viscosities inferred from models of postglacial rebound in the Patagonian icefield region^48^ are lower than our values as this region overlies the asthenospheric window associated with subduction of the Chile Rise, an active spreading centre^49^.

## Supplementary Information

Supplementary Information.

## Data Availability

Data and codes used in this study are available at: [https://github.com/danielmelnick/rocky-shore-Holocene-highstand].
