# Supplementary material for: Separating tectonic and climate signals in Holocene sea-level records using marine terraces in central Chile
Source: Sci Rep. 2026 Mar 13;16:9083. doi: 10.1038/s41598-026-43249-6 (PMC12992895; doi:10.1038/s41598-026-43249-6)
Supplement: Supplementary file 1 — Supplementary Information. [file 41598_2026_43249_MOESM1_ESM.pdf]

Supplementary materials

Separating tectonic and climate signals in Holocene sea-level records using marine terraces in central Chile

Daniel Melnick[1,2], Julius Jara-Muñoz[3], Ed Garrett[4], Gaëlle Plissart[1], Roland Freisleben[5], Manfred R. Strecker[5]

[1]*Instituto de Ciencias de la Tierra, Universidad Austral de Chile, Valdivia, Chile*
[2]*Millennium Institute of Oceanography, Concepción, Chile*
[2]*Faculty of Civil Engineering, Institute of Geo and environmental sciences, Hochschule Biberach, Germany*
[3]*Department of Environment and Geography, University of York, UK*
[4]*Institute of Geosciences, University of Potsdam, Germany*

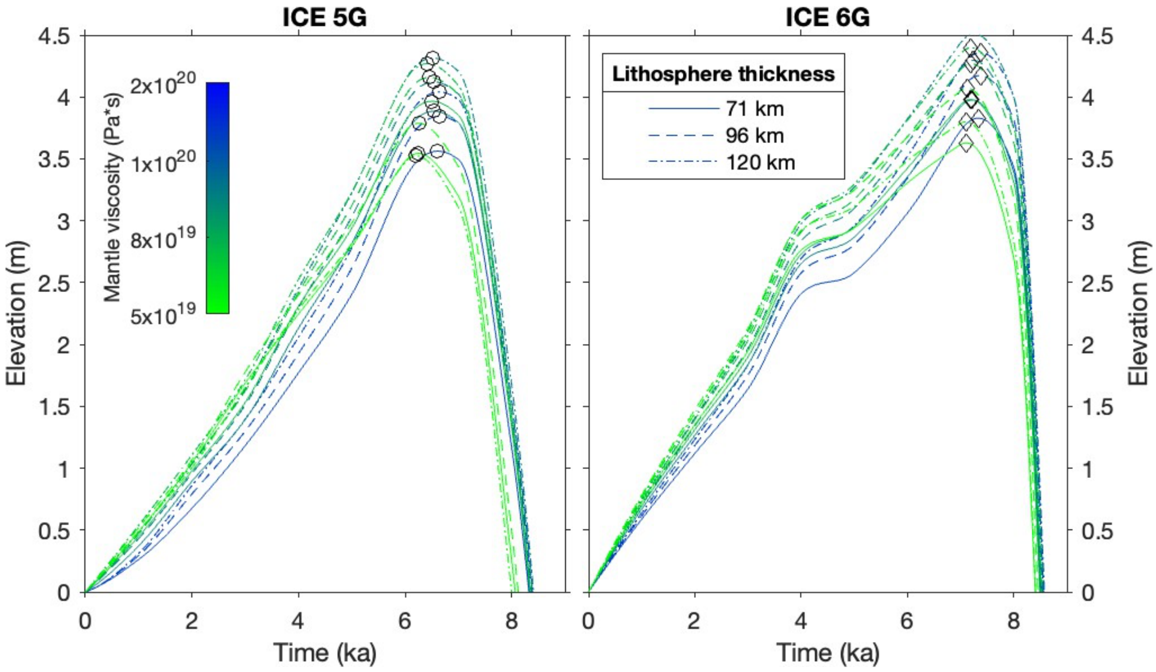

Figure S1. Sea-level curves predicted by the ICE-5G and 6G GIA models[1,2] for different Earth parameters used in this study.

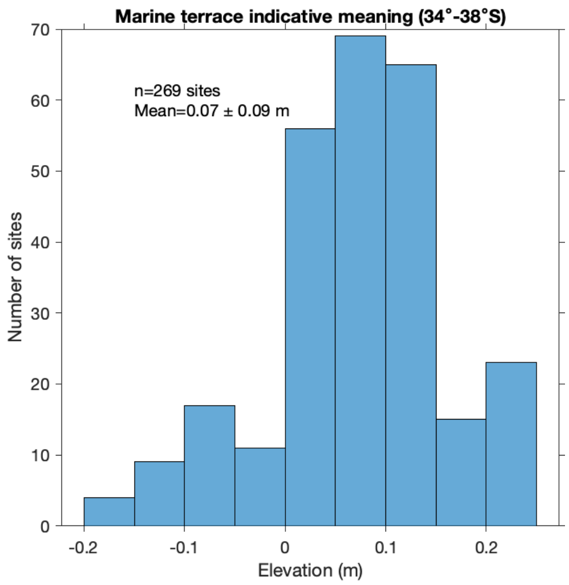

Figure S2. Histogram of indicative meaning estimates at marine terrace sites in central Chile using the method of Ref. [3].

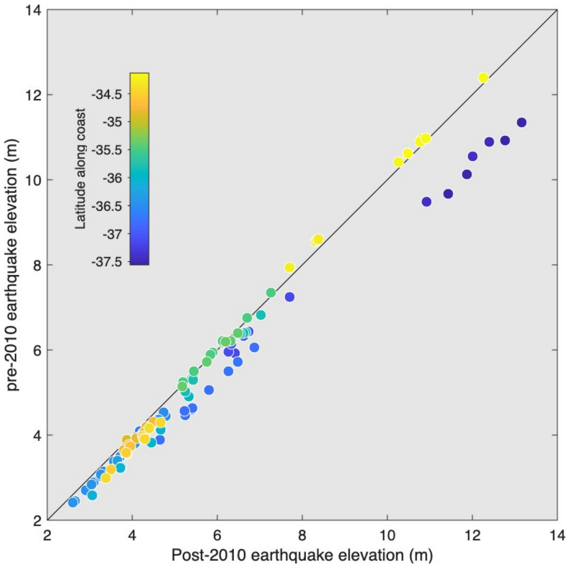

Figure S3. Elevation of Holocene shoreline angles corrected for coseismic land-level changes during the 2010 Maule earthquake using the model of Ref. [4].

References

1 Peltier, W. R. GLOBAL GLACIAL ISOSTASY AND THE SURFACE OF THE ICE-AGE EARTH: The ICE-5G (VM2) Model and GRACE. *Annual Review of Earth and Planetary Sciences* **32**, 111-149 (2004). https://doi.org:10.1146/annurev.earth.32.082503.144359

2 Peltier, W. R., Argus, D. F. & Drummond, R. Space geodesy constrains ice age terminal deglaciation: The global ICE-6G_C (VM5a) model. *Journal of Geophysical Research: Solid Earth* **120**, 450-487 (2015). https://doi.org:https://doi.org/10.1002/2014JB011176

3 Lorscheid, T. & Rovere, A. The indicative meaning calculator–quantification of paleo sea-level relationships by using global wave and tide datasets. *Open Geospatial Data, Software and Standards* **4**, 1-8 (2019).

4 Moreno, M. *et al.* Toward understanding tectonic control on the M w 8.8 2010 Maule Chile earthquake. *Earth and Planetary Science Letters* **321-322**, 152-165 (2012).
